# RNA-seq data of *Aspergillus tubingensis* NBRC 31125 in carbon catabolite repressor related to xylanase production

**DOI:** 10.1016/j.dib.2022.108700

**Published:** 2022-10-26

**Authors:** Ririn Krisnawati, Muhammad Nur Cahyanto, Sadjono Sardjono, Dian Anggaraini Suroto, Jaka Widada

**Affiliations:** a Department of Food and Agricultural Product Technology, Faculty of Agricultural Technology, Universitas Gadjah Mada, Yogyakarta, Indonesia; b Department of Agricultural Microbiology, Faculty of Agriculture, Universitas Gadjah Mada, Yogyakarta, Indonesia

**Keywords:** *Aspergillus tubingensis*, Glucose, RNA sequencing, Transcriptomics, Xylan, Xylanase

## Abstract

*Aspergillus tubingensis* NBRC 31125 is a prolific producer of endo-xylanase and β-xylosidase. However, the presence of glucose in the medium causes carbon catabolite repression (CCR) which inhibits the secretion of those enzymes. CCR in *Aspergillus* has been investigated in several ways. However, there are currently not any molecular data are available regarding the CCR of *A. tubingensis* in the xylan and glucose medium. Therefore, this research focuses on this aspect. The RNA of the strain was extracted in repressive condition, followed by sequencing using the Illumina NextSeq550 platform and reference assembly. The RNA-seq raw reads were submitted to the NCBI website's Sequence Read Archive database under the accession numbers SRR15412365 and SRR15412366, respectively. The data provide information for differentiating the response of xylanase and other enzymes production with and without glucose addition. The transcriptomics data can also be used to understand the xylan metabolism and CCR in *Aspergillus*

**Specifications Table**

**Table:** 

Subject	Food Science: Food Microbiology; Microbiology: Fungal Biology
Specific subject area	Transcriptomics of *Aspergillus tubingensis* with carbon catabolite repressor
Type of data	Table Database record Figure
How the data were acquired	RNA of fungal mycelium grown in xylan medium only and xylan medium containing glucose was extracted. RNA paired-end sequencing data was acquired by the next-generation sequencing Illumina NextSeq 550 platform. The quality of raw sequence reads was measured using FastQC v.0.11.9 and MultiQC v1.1. The sequence was assembled using the reference genome of *A. tubingensis* WU-2223L (GCF_013340325.1). The transcripts were quantified using the pseudo-alignment method by Kallisto v.0461. The data were visualised using T-REx v.2.0.
Data format	Raw sequence data in FASTQ format Analyzed data in table and figure
Description of data collection	Mycellium of *A. tubingensis* NBRC 31125 was collected from a liquid medium. The first medium contained xylan as the sole carbon source. Another medium contained xylan and glucose. Total RNA was extracted from mycelium after five days of incubation, followed by cDNA libraries construction and RNA sequencing using Illumina NextSeq 550
Data source location	Biotechnology Laboratory, Department of Food and Agricultural Product Technology, Faculty of Agricultural Technology, Universitas Gadjah Mada, Yogyakarta, Indonesia, with locations 7.768721N and 110.381900S
Data accessibility	FASTQ file of *A. tubingensis* NBRC 31125 grown in the xylan medium containing glucose and xylan medium alone can be found at Sequence Read Archive on the NCBI database website with the accession numbers SRR15412365 and SRR15412366, respectively. URL: http://www.ncbi.nlm.nih.gov/sra/?term=PRJNA753631

## Value of the Data

• The transcriptomics data on the effect of glucose addition in *A. tubingensis* NBRC 31125 grown in the xylan medium contribute to understanding the mechanism of carbon catabolite repression (CCR) related to xylanase at the molecular level.
• The transcriptomics data would be useful to researchers focusing on enzyme production using fungi and working on transcriptional regulation.
• The data obtained can be further analysed as the differentially expressed gene and transcript expression patterns of *A. tubingensis* NBRC 31125. This could lead to identification of genes related to CCR and future improvement of new strains of *A. tubingensis* through molecular engineering to increase the xylanase production
• The data can also be utilised for comparison with transcriptome analyses of other *Aspergillus* species or fungi under repressive conditions in order to identify predominant CCR patterns in fungi.

## 1 Data Description

**Fig. 1:**
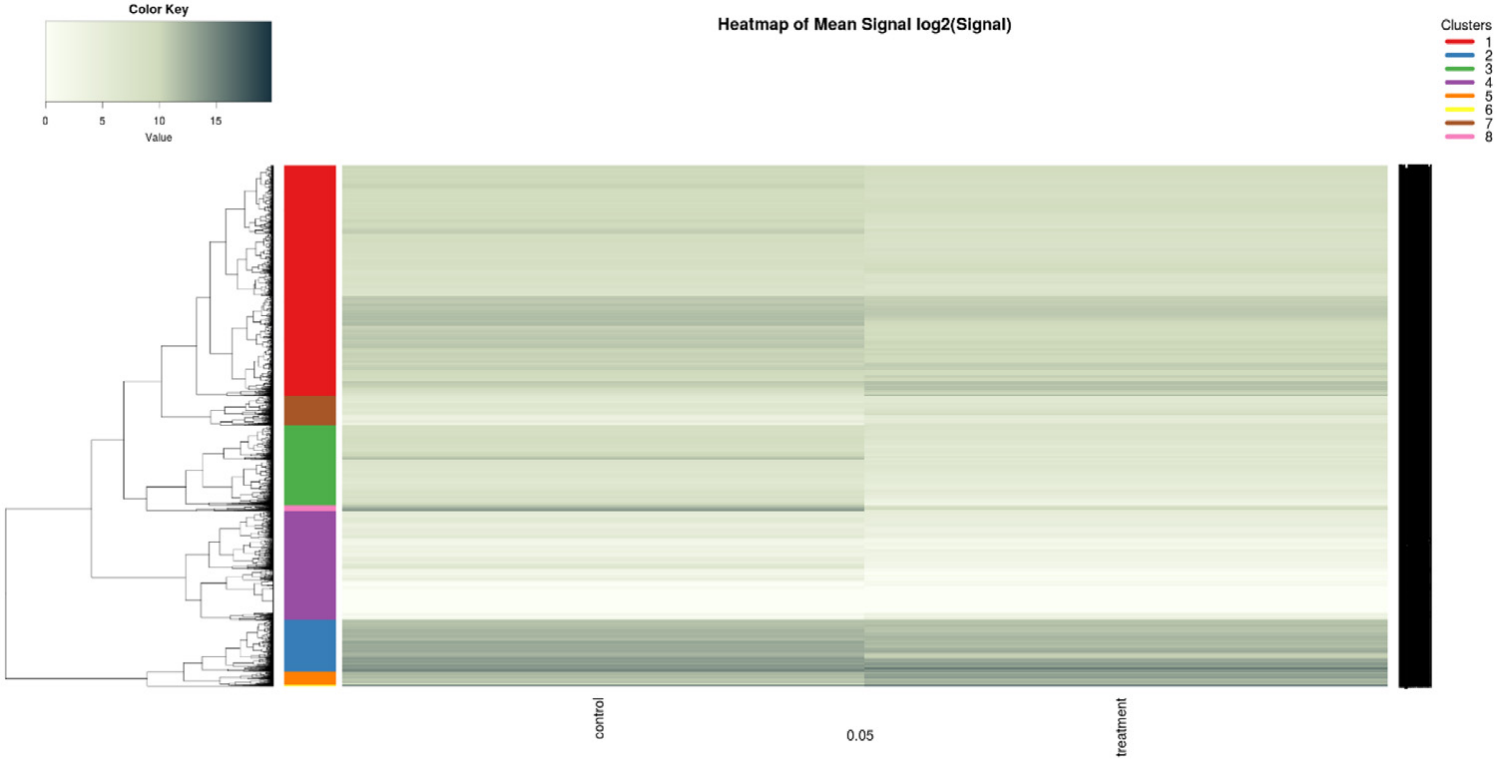
Heatmap of hierarchical clustering of gene transcription modulation patterns reported in *A.tubingensis* NBRC 31125 after growing on xylan (control) and xylan containing glucose medium (treatment).

The raw RNA sequencing data of *A. tubingensis* NBRC 31125, inoculated in the xylan medium containing glucose and xylan medium alone was stored in the FASTQ file and collected to the NCBI-SRA database with accession numbers SRR15412365 and SRR15412366, respectively. The descriptive RNA-seq data statistics of both samples are given in Table 1. Clean reads were mapped against the genome reference of *Aspergillus tubingensis* WU-2223L. Fig. 1 shows the difference in gene expression of *A. tubingensis* NBRC 31125 grown in xylan medium alone and xylan medium containing glucose in heatmap visualisation. The graph was constructed from the abundance estimates of raw transcripts of the fungi grown in such mediums. A dendrogram and colour bar were presented on the left side of the heatmap indicating the gene's clustering. The detail of the genes can be seen in Table S1 (supplementary data). The darker colour showed the higher gene transcription, conversely the lighter colour showed less transcription.

**Table 1:** The RNA-seq descriptive statistics data of *A. tubingensis* NBRC 31125 grown in the xylan and xylan containing glucose.

	Value	
Descriptive	*A. tubingensis* NBRC 31125 inoculated in xylan medium	*A. tubingensis* NBRC 31125 inoculated in xylan containing glucose medium
Total number of clean reads (bp)	32,017,693	34,061,555
Total number of clean bases (Gb)	9.6	10.2
Clean Reads Q30% (%)	85.8	85.8
GC Content (%)	52.0	51.5
Biosample ID	SAMN20702930	SAMN20702931
SRR number	SRR15412366	SRR15412365

Total number of clean reads: the number of the reads after filtering

The total number of clean bases: the amount of the bases after filtering.

Clean Reads Q30(%): the quality of bases with more than 30 values in clean reads GC Content (%): G&C base count is divided by the total base count.

## 2 Experimental Design, Materials and Methods

### 2.1 Cultivation

*Aspergillus tubingensis* NBRC 31125 was grown in the Potato Dextrose Agar at a temperature of 30°C for seven days. The fungi were inoculated in a liquid medium using techniques suggested by Chand et al., (2005) and Midorikawa et al. (2018) with slight modifications. A total of 1 mL inoculum containing 10^7^ spores/ml was inoculated on Erlenmeyer flasks containing 25 ml sterilised Mendel mineral salt consisting of urea (0.3 g/L), (NH_4_)_2_SO_4_ (1.4g/L), KH_2_PO_4_ (2.0 g/L), CaCl_2_.2H_2_O (0.4 g/L), MgSO_4_.7H_2_O (0.3 g/L), peptone (1.0 g/L), Tween 80 (0.2 g/L), FeSO_4_.7H_2_O (5.0 mg/L), MnSO_4_.7H_2_O (1.6 mg/L), ZnSO_4_.7H_2_O (1.4 mg/L), and CoCl_2_.6H_2_O (20.0 mg/L) at a pH 5.0, and 10g/L of xylan from a corn cob (Sisco) . A total of 50 g/L glucose was added to the xylan medium to induce a repressive condition. Erlenmeyer flasks were incubated at a temperature of 30°C for five days [1], [2], [3]. After incubation, the mycelium was collected for RNA extraction. All experiments were performed in duplicate.

### 2.2 Extraction and Sequencing of RNA

Total RNA was extracted from the mycelium of both samples (duplicate) using Direct-Zol RNA Miniprep Plus (Zymo Research) according to the manufacturer's instructions. RNA concentration and quality were determined by Nanodrop Spectrophotometer and Agilent 2100 Bioanalyser. The performed works were rRNA removing, RNA fragmentation, double-stranded cDNA synthesis, adenylated-end addition, adapter addition, PCR amplification, library-quality test, and sequencing on next-generation sequencing NextSeq550 platforms.

### 2.3 Data workflow of RNA Sequencing

The clean reads were obtained by removing the raw reads of RNA-seq with adaptors. The quality clean reads were analysed using the software FastQC v.011.9 and MultiQC v1.1 [4,5]. The reads were stored in the FASTQ file. The data was mapped into *Aspergillus tubingensis* WU-2223L genome (GCF_013340325.1) as a genome reference [6]. Kallisto v.0461 was used to quantify the abundance estimates of raw transcripts [7]. Data visualisation was performed using T-REx v2.0 [8].

## Ethics Statements

Not applicable.

## CRediT authorship contribution statement

**Ririn Krisnawati:** Investigation, Formal analysis, Data curation, Writing – original draft. **Muhammad Nur Cahyanto:** Project administration, Funding acquisition, Supervision, Writing – review & editing. **Sadjono Sardjono:** Resources. **Dian Anggaraini Suroto:** Supervision. **Jaka Widada:** Supervision, Conceptualization, Methodology, Validation, Writing – review & editing.

## Declaration of Competing Interest

The authors declare that they have no known competing financial interests or personal relationships that could have appeared to influence the work reported in this paper.

## Data Availability

Raw reads of A. tubingensis NBRC 31125 grown in xylan and xylan containing glucose medium (Original data) (DIB).

## Appendix Supplementary materials

Image, application 1
